# Association of interleukin 7 receptor gene polymorphism rs6897932 with multiple sclerosis patients in Khuzestan

**Published:** 2014-07-04

**Authors:** Nastaran Majdinasab, Mahshid Hosseini Behbahani, Hamid Galehdari, Maryam Mohaghegh

**Affiliations:** 1 Musculoskeletal Rehabilitation Research Center AND Department of Neurology, School of Medicine, Ahvaz Jundishapur University of Medical Sciences, Ahvaz, Iran; 2 Department of Biochemistry, Payame Noor University, Tehran, Iran; 3 Departmanet of Genetic, School of Sciences, Shahid Chamran University, Ahvaz, Iran

**Keywords:** Multiple Sclerosis, Interleukin 7 Receptor, Polymorphism, Amplified Refractory Mutation System, Relapsing-Remitting Multiple Sclerosis

## Abstract

**Background: **Multiple sclerosis (MS) is a chronic inflammatory demyelinating and neurodegenerative disease of central nervous system with unknown causes. Etiology of MS involves both genetic and environment factors. The interleukin 7 receptor (IL7R) gene is a promising candidate for MS, because its involvement in the autoimmunity, regulation of the T-cell homeostasis, proliferation, and anti-apoptotic signaling.

**Methods:** We investigated the association of the IL7R gene polymorphism rs6897932 in MS patients in a case and control study. In this case and control study participating, 127 relapsing-remitting MS (RRMS) patients (mean age: 32.25, age range: 16-57) selected according McDonald criteria, and 109 ethnically, sex and age matched healthy control (mean age: 27.44, age range: 14-63) with no personal or family history of autoimmune diseases were studied. DNA was extracted from whole blood using high pure polymerase chain reaction template preparation kit from Roch Company. Amplification refractory mutation system method was applied to define the genotyping C/T within exon 6 of the IL7R gene among individuals.

**Results: **Evaluation of the IL7R gene polymorphism revealed that the T allele and the C/T and T/T genotypes are present in 53.5%, 42.5%, 4.0%, and 68.8%, 26.6%, 4.6% in MS patients and controls, respectively. Comparison between alleles and genotypes in the MS patients and healthy controls show significant differences (P = 0.038).

**Conclusion: **The distribution of the rs6897932 polymorphism is significantly different in our case/control study in Khuzestan Province. This single nucleotide polymorphism causes alternative splicing in exon 6 of the IL7R gene with possible influence of the autoimmunity.

## Introduction

Multiple sclerosis (MS) is a disabling disorder affecting, especially young women.^1^ It is a disease of the central nervous system (CNS) characterized by multiple regions of demyelization and inflammation along axons with a T-cell-mediated autoimmune etiology.^2^ Although the cause of MS is unknown, but both genetic and environmental factors play important roles.^3^ New technologies identified genetic polymorphisms associated with MS susceptibility among which immunologically relevant genes are significantly overrepresented.^4^

Until 2007, only one inherited risk factor had been validated across multiple populations, the major histocompatibility complex (MHC) class II DRB1p1501 allele, which confers a 3- to 4-fold relative risk, and implicates Antigen presentation to CD4 T-cells in MS pathogenesis.^5^ The advent of whole-genome screening tools has now enabled the discovery of new MS risk genes, including interleukin 7 receptor (IL7R) and interleukin 2 receptor A gene (IL-2RA), CD58, and CLEC16A.^6^ In 2007, the first genome-wide association study (GWAS) for MS susceptibility was reported. This first-pass follow-up resulted in the identification of three strongly associated single nucleotide polymorphisms (SNPs) outside of the MHC, namely rs6897932 in the IL7R A gene (IL7RA) and both rs12722489 and rs2104286 within the interleukin 2 receptor A gene (IL2RA).^7^ These associations were replicated by a number of groups and further refined in subsequent analyses.^8^

IL7R alpha (IL7Rα) (CD127) is an essential pleiotropic receptor in immunology. IL7Rα functions as a receptor for two signaling cascades: IL7 and thymic stromal lymphopoietin. In the IL7 pathway, IL7 interacts with IL7Rα and the gamma chain (γc) (CD132), forming the signaling complex.^9^ The IL7/IL7RA interaction is important for the maintenance of memory T-cells and the development, proliferation, and survival of B- and T-cells, especially CD+ T-cells, which are present in inflammatory lesions of MS patients.^10^

Although individual genes contribute only a small part to MS susceptibility, they might be used as biomarkers, thus helping to identify accurate diagnosis, predict clinical disease course, and response to therapy.^4^ In this study, we aimed to determinate the distribution of the rs6897932 SNP in MS patients compared to healthy controls in Khuzestan Province.

## Materials and Methods

***Sample collection***

A total of 127 unrelated relapsing-remitting MS (RRMS) patients from Khuzestan Province, Iran (103 women and 24 men, mean age was 32.2 years, and age range was 16-57) were collected. The diagnosis of MS was made according to the McDonald criteria.^11^ Furthermore, we included 109 unrelated Khuzestan healthy controls in this study (92 women and 17 men, mean age was 27.4, and age range was 14-63).

***Genotyping***

Polymerase chain reaction (PCR) with allele specific primers (amplification refractory mutation system [ARMS] primers) was used to detect the rs6897932 polymorphism at exon 6. Four primers designed, two of them for internal control and two specific for T or C allele. For this polymorphism in exon 6, one sequence-specific forward primers: 5′-AAGAAGGGAAGAGAGCATTGG-3′, and three sequence-specific reverse primers that two of them: 5′-GAAAAAACTCAAAATGCTGATGG-3′ (for C allele) and 5′-AGAAAAAACTCAAAATGCTGATGA-3′ (for T allele) and one reverse were primer for internal control 5′-TTACTTTGGGGACAGCGTTT-3′ used.

***DNA extraction***

Peripheral blood from patients and controls was collected in ethylenediaminetetraacetic acid tube and genomic DNA was extracted and purified from whole blood lymphocytes by high pure PCR template preparation kit from Roch Company, Germany according to the manufacturer’s instructions. Demographic and clinical profiles of MS patients and controls are shown in table 1. Each combination of forward and C or T reverse primers contained 301 bp fragment and 577 bop fragment for internal control. DNA was amplified by one rounds of PCR performed with 3 μl of DNA in a 25 μl reaction mixture containing 12.5 master mix ampliqon, 1 μM of forward primer and reverse for internal control and 1.5 for C primer and 2 for T primer. Thermal cycling conditions were as follows: denaturation of 94° C for 5 min followed by 35 cycles of denaturation at 94° C for 30 s, annealing at 62° C for 30 s and extension at 72° C for 30 s. The amplification was followed by a final extension step at 72° C for 5 min. PCR product viewed by agarose gel 1.5% and it been seen with gel doc.

***Statistical analysis***

To examine the susceptible *rs6897932 *polymorphisms in MS disease, χ^2^ was calculated and Fisher’s exact test was accomplished using SPSS for Windows 15.0 (SPSS Inc., Chicago, IL, USA). P <0.05 was regarded as significant.

## Results

In this study, 127 RRMS patients and 109 healthy controls were compared regarding the rs6897932 polymorphism within the IL7R gene by ARMS-PCR. Allele and genotype frequency in patient and control group were significantly different. In this study, we evaluated and compared this SNP genotyping in 127 patients with 109 healthy control using ARMS method. Results show that rs6897932 is significantly different in and controls, while compared genotypes based on gender has shown different results. Although T/T genotype has not seen in men suffering MS, it is 4 (3.6%) in the control group.

**Table 1:** Demographic and clinical profiles of multiple sclerosis patients and controls

**Variable**	**Female/male, n (%)**	**Age (years), mean ± SD**	**Age range (years)**	**Age at onset (years), (mean ± SD)**
MS patients	103 (81.1)	32.25 ± 7.80	16-57	27.57 ± 6.80
	24 (18.9)			
Controls	92 (84.4)	27.44 ± 7.00	20-63	-
	17 (15.6)			

MS: Multiple sclerosis; SD: Standard deviation

On the other hand, this genotype is 4 (3.9%) cases in women, while there was no record in control women. The frequency of rs6897932 genotype in Khuzestan MS patients and controls is shown in table 2 and according to gender is shown in table 3.

**Table 2:** The frequency of rs6897932 allele and genotype in Khuzestan multiple sclerosis patients and controls

	**Patients**	**Controls**	**P**
Allele	n = 254	n = 218	
C	183	172	0.047
T	61	36	
Genotype	n = 127	n = 109	
C/C	66	73	0.029
C/T	51	26	
T/T	5	5	

**Table 3:** The frequency of rs6897932 genotype in Khuzestan multiple sclerosis patients and controls according to gender

**Genotype**	**MS patients**	**Control**
CC		
Male	10	8
Female	58	68
CT		
Male	14	5
Female	40	24
TT		
Male	0	4
Female	5	0

MS: Multiple sclerosis

## Discussion

MS incidence rates vary significantly depending on geographic location and ethnic origin. Recent studies indicate a relatively high prevalence of MS in the Iranian population. The reason for this increasing prevalence remains elusive.^12^

The inflammatory lesions typical of multiple sclerosis show autoimmune features and depend partly on genetic factors.^13^ Although the pathogenesis of MS has yet to be elucidated, there is increasing evidence that MS is the result of an interaction of genetic and environmental factors.^14^ While MS genome screens have not identified a single MS susceptibility locus, multiple chromosomal regions showing suggestive linkage have been observed.^15^ Indicating that MS susceptibility may be determined by the interaction of multiple genes exerting modest effects.^16^ The best-established region implicated in predisposition to MS is the major histocompatibility complex on chromosome 6p21, specifically the human leukocyte antigen (HLA)-DRB1* 1501 class II allele.^17^ However, it explains less than 50% of the total genetic basis of the disease.^18^ In addition to HLA, several genes have been shown to be associated with the risk and progression of MS including genes involved in antigen presentation, cytokine genes, and T-cell receptor genes.^19^ Seven GWAS have led to the identification of around 15 validated non-HLA risk loci for MS, including among others IL2RA, IL7R, CD58, EVI5, and CD40.^20^ The HLA allele and the IL7RA allele are both great examples of common alleles.^21^ Ahmadzadeh et al. in their study demonstrated the effects of some SNPs on the IL7Rα protein in Iranian MS patients.^22^ IL7 is an essential cytokine for the development and homeostatic maintenance of T and B lymphocytes. Binding of IL7 to its cognate receptor, the IL7R, activates multiple pathways that regulate lymphocyte survival, glucose uptake, proliferation, and differentiation.^23^

Both GWAS data and independent study simultaneously reported the association of SNP (rs6897932) in the IL7RA with MS.^24^ Latter study provided evidence for the functional impact of this SNP (T244I) on gene expression.^7^ The rs6897932 affects alternative splicing of exon 6, leading to increased skipping of the exon. This is predicted to increase production of soluble IL7Rα chain for individuals carrying the risk allele atrs6897932. The alternative splicing of IL7R has potent consequences for the function of the receptor, as transcripts that include exon 6 encode a membrane-bound IL7Rα, whereas transcripts that skip exon 6 encode a soluble form of the protein. Carriers of the “C” allele at rs6897932 produce less membrane-bound IL7Rα protein compared with carriers of the “T” allele, leading to a further increase of the soluble form of IL7Rα.^18^

We found an association between rs6897932 polymorphism and RRMS patients in Khuzestan by comparing this SNP in patient and control groups. We suggest this SNP as one of various genetic predisposing for MS patients, at least in Khuzestan Province.

## Conclusion

The aim of this study was to identify single nucleotide polymorphisms in the IL7Rα chain gene in MS patients from Khuzestan Province. We used ARMS technique for genotyping of our samples. The influence of the rs6897932 within exon 6 of the IL7RA gene on MS susceptibility is replicated in a number of studies.^7^

In our study, the frequency of T allele was significantly higher in MS patients than controls. CT genotype is probably associated with the MS disease, what need to confirm with large sample size.

## Notes

**How to cite this article: **Majdinasab N, Hosseini Behbahani M, Galehdari H, Mohaghegh M. Association of interleukin 7 receptor gene polymorphism rs6897932 with multiple sclerosis patients in Khuzestan. Iran J Neurol 2014; 13(3): 168-71.
